# Flower-like PEGylated MoS_2_ nanoflakes for near-infrared photothermal cancer therapy

**DOI:** 10.1038/srep17422

**Published:** 2015-12-03

**Authors:** Wei Feng, Liang Chen, Ming Qin, Xiaojun Zhou, Qianqian Zhang, Yingke Miao, Kexin Qiu, Yanzhong Zhang, Chuanglong He

**Affiliations:** 1 College of Chemistry, Chemical Engineering and Biotechnology, Donghua University, Shanghai 201620, China; 2 College of Materials Science and Engineering, Donghua University, Shanghai 201620, China

## Abstract

Photothermal cancer therapy has attracted considerable interest for cancer treatment in recent years, but the effective photothermal agents remain to be explored before this strategy can be applied clinically. In this study, we therefore develop flower-like molybdenum disulfide (MoS_2_) nanoflakes and investigate their potential for photothermal ablation of cancer cells. MoS_2_ nanoflakes are synthesized via a facile hydrothermal method and then modified with lipoic acid-terminated polyethylene glycol (LA-PEG), endowing the obtained nanoflakes with high colloidal stability and very low cytotoxicity. Upon irradiation with near infrared (NIR) laser at 808 nm, the nanoflakes showed powerful ability of inducing higher temperature, good photothermal stability and high photothermal conversion efficiency. The *in vitro* photothermal effects of MoS_2_-PEG nanoflakes with different concentrations were also evaluated under various power densities of NIR 808-nm laser irradiation, and the results indicated that an effective photothermal killing of cancer cells could be achieved by a low concentration of nanoflakes under a low power NIR 808-nm laser irradiation. Furthermore, cancer cell *in vivo* could be efficiently destroyed via the photothermal effect of MoS_2_-PEG nanoflakes under the irradiation. These results thus suggest that the MoS_2_-PEG nanoflakes would be as promising photothermal agents for future photothermal cancer therapy.

Photothermal therapy (PTT), as a non-invasive and potentially efficient cancer therapy, has attracted significant attention in recent years^1^,^2^. PTT based on photo-absorbing nanomaterials has been suggested as an alternative procedure to the conventional approaches such as surgery, radiation therapy and chemotherapy^3^,^4^. In a typical PTT, the use of near-infrared (NIR) light in the range of 700–1100 nm for the induction of hyperthermia is highly attractive due to its high transparency in biological tissue, blood and water^5^,^6^. An ideal photothermal agent can efficiently transfer the absorbed NIR light into heat without causing toxic side effects, is thus a prerequisite for successfully PTT^7^,^8^.

To address this, a variety of well-designed NIR-absorbing photothermal agents have been extensively investigated for photothermal cancer therapy with varying success, but still far from the optimal. Noble metal nanomaterials with various morphologies, such as Au nanoparticles^9^, Au nanoshells^10^, Au nanocages^11^, Au nanorods^12^, Ag nanospheres^13^, Ge nanocrystals^14^, and Pd nanosheets^15^, exhibit relatively high photothermal conversion efficiency due to their unique surface plasmon resonance (SPR) properties, but their relatively high cost has restricted their wide use^16^. Carbon-based nanomaterials, including carbon nanotube^3^,^17^,^18^,^19^,^20^ and graphene^21^,^22^, have also demonstrated promising photothermal properties, but certain limitations also exist, such as easy photobleaching, poor hydrophilicity and/or unsatisfactory photothermal conversion efficiency^23^. The other newly emerging photothermal agents such as semiconductor nanomaterials^16^,^24^,^25^ and conjugated polymers^5^,^7^,^26^,^27^,^28^, which have also shown great potential for photothermal treatment, but their potential long-term toxicity and photothermal conversion efficiency remain central concerns for clinical applications^16^,^29^.

Recently, the two-dimensional transition metal dichalcogenides (TMDC) nanosheets such as MoS_2_^30^ and WS_2_^31^, have emerged as novel alternative photothermal agents with encouraging early results. Among them, MoS_2_ has attracted tremendous interests in a wide variety of fields including nanoelectronics, sensor and catalysis^32^,^33^,^34^. More recently, the biomedical applications of MoS_2_ have been reported and expanded rapidly because of its good biocompatibility and high photothermal performance. Chou *et al.* first demonstrated the effectiveness of using single-layer chemically exfoliated MoS_2_ (ceMoS_2_) sheets as a novel NIR photothermal agent for PTT, which exhibited greater absorbance in the NIR region than that of both graphene oxide (GO) and gold nanorods^35^. Benefiting from its large surface area and high NIR absorbance, a variety of MoS_2_-based theranostic agents have been created by integrating different theranostic modalities into a single nanoplatform for combined cancer treatments with real-time diagnosis^23^,^30^,^36^,^37^,^38^,^39^. However, most of the above-mentioned reports were based on the chemically exfoliated two dimension (2D) single layer MoS_2_ nanosheets, which require complex fabrication process and are difficult to control the size (size distribution usually from several nanometers to micrometers) and thickness of nanosheets^23^. Very few of them lead to MoS_2_ materials with a designed narrow particle size distribution, especially kept the size in the range of 50 to 300 nm, which strictly demanded in application of blood stream or drug delivery systems^40^,^41^. As a matter of fact, the use of MoS_2_ nanomaterials in the biomedical field is still in its infancy. Therefore, it is of great interest to develop novel MoS_2_-based photothermal agents with high photothermal conversion performance and excellent biocompatibility.

Herein, we report the development of PEGylated MoS_2_ nanoflakes (denoted as MoS_2_-PEG) with three-dimensional flower-like morphology, which sever as an effective photothermal agent under NIR laser irradiation (808 nm). The flower-like MoS_2_ nanoflakes with uniform morphology were synthesized via a facile one-pot solvothermal method and then modified with lipoic acid-terminated polyethylene glycol (LA-PEG), to improve their colloidal stability and biocompatibility. The obtained MoS_2_-PEG nanoflakes were well-characterized, and their NIR photothermal conversion efficiency, photothermal stability and biocompatibility were also evaluated.

## Results and Discussion

### Synthesis and characterization of MoS_2_-PEG nanoflakes

**Figure 1:**
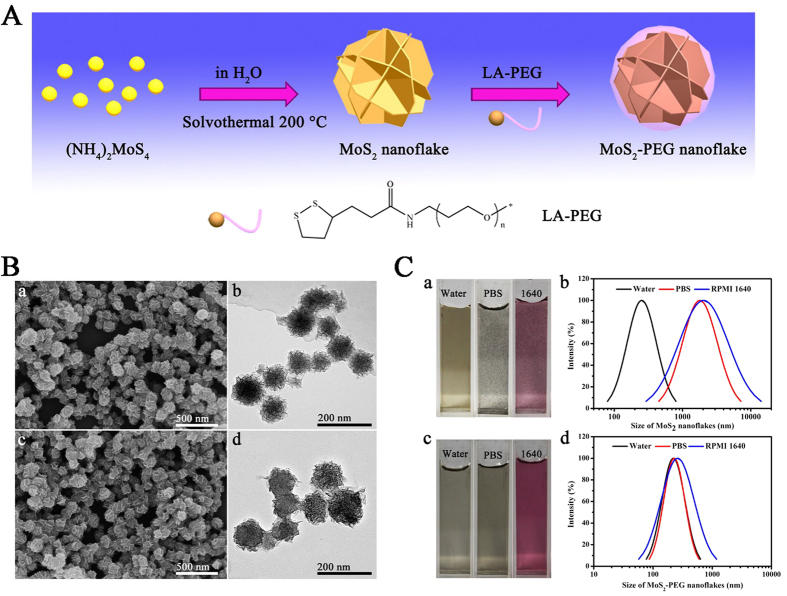
(**A**) Schematic illustration of the synthetic route for MoS_2_-PEG nanoflakes. (**B**) (a) TEM and (b) FESEM images of MoS_2_ nanoflakes; (c) TEM and (d) FESEM images of MoS_2_-PEG nanoflakes. (**C**) Photos of (a) MoS_2_ and (c) MoS_2_-PEG nanoflakes in DI water, PBS and cell culture medium (RPMI 1640) containing 10% fetal bovine serum (FBS). The hydrodynamic diameters of (b) MoS_2_ and (d) MoS_2_-PEG nanoflakes in DI water, PBS and RPMI 1640 medium containing 10% FBS determined by using dynamic light scattering (DLS) measurement.

The preparation of MoS_2_-PEG nanoflakes is schematically illustrated in the Fig. 1A. The MoS_2_ nanoflakes were firstly synthesized by a simple hydrothermal method, and then functionalized by immobilizing the pre-synthesized LA-PEG polymer onto their surface to construct the MoS_2_-PEG nanoflakes. Notably, the two sulfur atoms in the LA unit enabled much stronger binding to MoS_2_ nanoflakes compared to the employ of a single thiol^30^, therefore LA-PEG could firmly graft onto the surface of MoS_2_ nanoflakes.

The typical field emission scanning electron microscopy (FESEM) and transmission electron microscopy (TEM) images in Fig. 1B provide direct evidence for the formation of the final products. The SEM image in Fig. 1Ba shows that the synthesized MoS_2_ nanoflakes have spherical nanoflower-like structure with an average diameter of around 90 nm, while the TEM image in Fig. 1Bb reveals that these MoS_2_ nanoflowers are composed of numerous well-defined nanosheets. From the SEM image in Fig. 1Bc, the functionalized nanoflakes did not differ significantly from their unmodified counterparts in shape, indicating that only a thin organic layer was coated on the surface of the nanoflakes, which can be further corroborated by the TEM image in Fig. 1Bd.

The stability of PTT agents under physiological conditions is a critical issue relating to their biomedical application^30^. Although the prepared MoS_2_ nanoflakes were initially well dispersed in Deionized (DI) water, they would aggregate and precipitate in phosphate buffer saline (PBS) and cell culture medium containing 10% fetal bovine serum (FBS) (Fig. 1Ca), which is consistent with the previous reports on the MoS_2_ nanosheets^30^. The change in stability of the MoS_2_ nanosheets before and after PEGylation was also determined by dynamic light scattering (DLS) measurement. As shown in Fig. 1Cb, the average hydrodynamic size of the naked MoS_2_ nanoflakes in water is 253 nm. However, when they were separately dispersed in the PBS and cell culture medium containng 10% FBS, their hydrodynamic sizes were respectively increased to 1796 and 2035 nm as a result of the aggregation of the nanoflakes (Fig. 1Cb). In contrast, the PEGylated MoS_2_ nanoflakes displayed a remarkably enhanced physiological stability and were well dispersed in water, PBS and cell culture medium without any agglomeration even after 24 h storage (Fig. 1Cc). The PEGylation lead to a slight increase in hydrodynamic size of the MoS_2_-PEG nanoflakes when they were dispersed in PBS and Roswell Park Memorial Institute (RPMI) 1640 with 10% FBS, as evidenced in Fig. 1Cd. This may be due to the adsorption of some proteins onto the surface of nanoflakes^42^,^43^.

**Figure 2:**
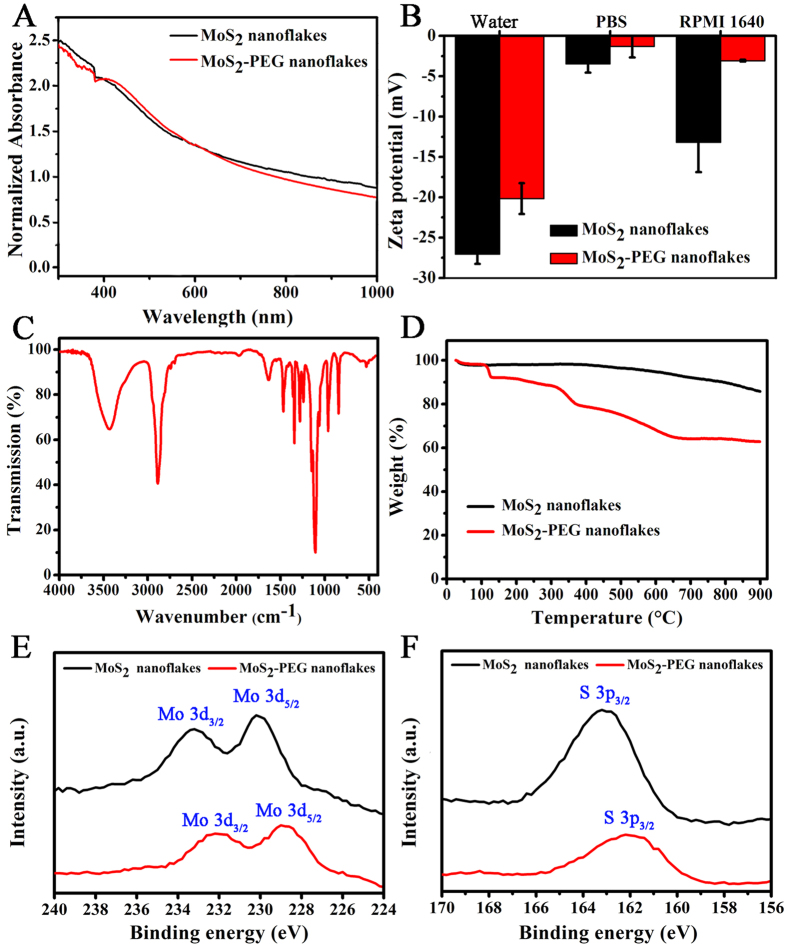
(**A**) UV-vis absorption spectra of the MoS_2_ and MoS_2_-PEG nanoflakes solution. (**B**) Zeta potentials of MoS_2_-PEG nanoflakes in distilled water, PBS buffer and RPMI 1640 medium containing 10% FBS. (**C**) FTIR spectrum of MoS_2_-PEG nanoflakes. (**D**) TG curves of MoS_2_ and MoS_2_-PEG nanoflakes. (**E**) XPS spectra of Mo 3d orbits. (**F**) XPS spectra of S 2p orbits.

The Ultraviolet-visible (UV-vis) absorbance spectra (Fig. 2A) reveal that both MoS_2_ and MoS_2_-PEG had a similar strong absorbance from UV to NIR regions, suggesting that the PEGylation did not alter the spectral characteristics of MoS_2_ nanoflakes. Figure 2B indicates the difference in zeta potential of both nanoflakes dispersed in different medium. It was observed that both MoS_2_ and MoS_2_-PEG nanoflakes were highly negatively charged when dispersed in DI water, revealing well-dispersion of these nanoflakes in water^38^. However, the zeta potential of nanoflakes decreased after PEGylation regardless of the dispersing medium, which is attributed to the charge shielding effect of the incorporated PEG layer^44^,^45^. Among the different dispersing medium, the nanoflakes dispersed in PBS have the lowest absolute zeta potential, which may result from the rapid nanoflakes aggregation in the presence of salts. While the nanoflakes dispersed in RPMI 1640 culture medium with 10% FBS exhibit a reduced zeta potential than those dispersed in DI water, which is due to that some albumen in the medium was absorbed on the nanoflakes surface^46^. The presence of PEG on the MoS_2_-PEG nanoflakes was further confirmed by Fourier transform infrared (FTIR) analysis. As seen from the spectrum of MoS_2_-PEG in Fig. 2C, the broad bands between 3700 and 3000 cm^−1^ are attributed to the O-H stretching from the intermolecular and intramolecular hydrogen bonds^47^. The absorptions at 1627, 1427, 1112, 875 and 607 cm^−1^ are attributed to MoS_2_^30^,^48^,^49^, and the characteristic vibrational bands at 3000–2800 cm^−1^ can be assigned to the stretching of the C-H alkyl stretching band in PEG. Thus, the FTIR results further confirmed the successful PEGylation of MoS_2_ nanoflakes. The presence of LA-PEG was further proven by Thermogravimetric (TG) analysis. From the TG curves (Fig. 2D), the overall amount of coated LA-PEG could be calculated. When the temperature was increased to about 900 °C, the weight loss values of MoS_2_ nanoflakes and MoS_2_-PEG were 14.2% and 37.2%, respectively. As a result, the amount of coated LA-PEG on the surface of MoS_2_ nanoflakes was about 23.0%. The chemical bonding information of the prepared nanoflakes can be investigated by The X-ray photoelectron spectroscopy (XPS) analysis. The XPS spectra of the Mo 3d and S 2p regions can be shown in Fig. 2E,F, respectively. The peaks correlating to Mo^4+^ (in the positions of 233.2 and 230.2 eV for MoS_2_ nanoflakes, as well as at 232.2 and 229 eV for MoS_2_-PEG nanoflakes (Fig. 2E), and the peaks corresponding to S^2−^ (in the position of 163.2 eV for MoS_2_ nanoflakes, and at 162.2 eV for MoS_2_-PEG nanoflakes (Fig. 2F) can be seen in the spectra of MoS_2_ and MoS_2_-PEG nanoflakes, respectively. It can be noted that the positions of Mo (3d_3/2_), Mo (3d_5/2_) and S (2p_3/2_) for MoS_2_ nanoflakes shifted to lower binding energy after PEG modification. This may be influenced by the presence of PEG grafting^23^.

### *In vitro* photothermal performance of MoS_2_-PEG nanoflakes

**Figure 3:**
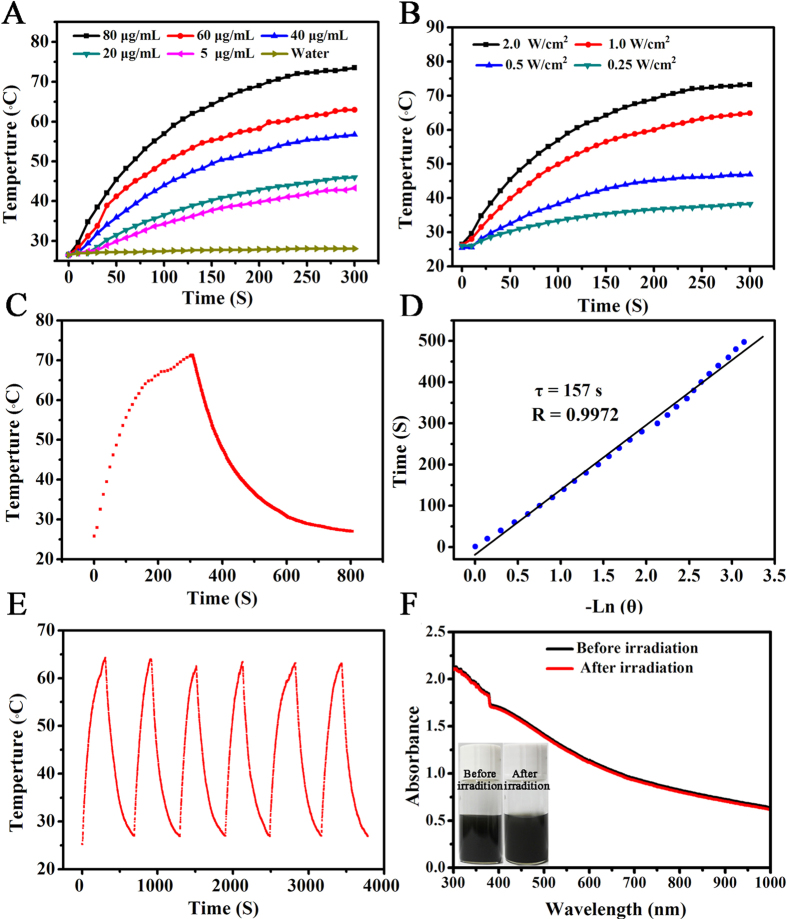
(**A**) Temperature profiles of pure water and MoS_2_-PEG nanoflakes dispersions with different concentrations (5, 20, 40, 60 and 80 μg/mL) as a function of 808-nm laser irradiation time for 5 min at a power density of 2 W/cm^2^. (**B**) Photothermal heating curves of MoS_2_-PEG nanoflakes solution at the concentration of 80 μg/mL under 808-nm laser irradiation at various power densities (0.25, 0.5, 1.0 and 2.0 W/cm^2^) for 5 min. (**C**) Photothermal effect of an aqueous dispersion of MoS_2_-PEG nanoflakes solution irradiated by using an 808-nm laser at a power density of 2 W/cm^2^. The laser was turned off after irradiation for 300 s. (**D**) Plot of cooling time (after 300 s) versus negative natural logarithm of the driving force temperature obtained from cooling stage as shown in (**C**). The time constant (τ_s_) for heat transfer of the system is determined to be 157 s. (**E**) Temperature monitoring of a MoS_2_-PEG nanoflakes solution at the concentration of 60 μg/mL during for successive six cycles of an on-and-off laser. (**F**) UV-vis absorption spectra of the MoS_2_-PEG nanoflakes solution before and (right) after NIR 808-nm irradiation at the power of 2 W/cm^2^ for successive six cycles of an on-and-off laser irradiation. The insets show that digital photographs of the MoS_2_-PEG nanoflakes solution (left) before and (right) after irradiation.

The photothermal performance of the MoS_2_-PEG nanoflakes was first examined by monitoring the temperature increase of 0.4 mL aqueous solution containing various concentrations of MoS_2_-PEG nanoflakes under NIR laser irradiation (λ = 808 nm, 2 W/cm^2^). As shown in Fig. 3A, significant temperature increases were recorded for various concentrations of MoS_2_-PEG nanoflakes under laser irradiation, and the rate of temperature increase was more pronounced within the initial 3 min of laser irradiation. The solution containing 80 μg/mL of MoS_2_-PEG nanoflakes indicates a maximum temperature increase from 26.5 to 73.5 °C within 5 min, while pure water without nanoflakes records a negligible temperature change of 1.6 °C. Such a photothermal effect of MoS_2_-PEG nanoflakes is enough to induce thermal damage to the targeted tissue, making them promising potential photothermal agents. Figure 3B shows the temperature increases of the solution containing 80 μg/mL of the MoS_2_-PEG nanoflakes after laser radiation at various power densities. It was found that the solution temperature was markedly increased with the increase of power densities, indicating an obvious laser-power-dependent photothermal effect for MoS_2_-PEG nanoflakes. After the laser irradiation at 2 W/cm^2^, the solution temperature was rapidly increased to 73.2 °C within 5 min. To further assess the photothermal transduction ability of MoS_2_-PEG nanoflakes, the solution containing 80 μg/mL of the MoS_2_-PEG nanoflakes was exposed to NIR laser at 2 W/cm^2^ for 5 min, and then the laser was turned off. Figure 3C shows the typical photothermal profile of MoS_2_-PEG nanoflakes. The rapid cooling of the solution after the laser was turned off revealed that the MoS_2_-PEG nanoflakes solution had a good thermal conductivity. Thus, according to the data obtained (Fig. 3C,D) and the methods reported previously, the photothermal conversion efficiency of the synthesized MoS_2_-PEG nanoflakes was calculated to be 27.6% (Supplementary Information), which is slightly higher than that of the recently reported MoS_2_-chitosan (MoS_2_-CS) nanosheets (24.4%)^38^. To further demonstrate the photostability of MoS_2_-PEG nanoflakes, periodic laser on/off control with 808 nm NIR light were used, in which the solution of MoS_2_-PEG nanoflakes was irradiated under NIR laser for 5 min, followed by naturally cooling down to the room temperature without NIR laser irradiation. As shown in Fig. 3E, after 6 cycles of laser on/off irradiation, no notable decrease for the temperature elevation was observed during the experiment. Furthermore, from Fig. 3F, the UV-vis spectrum of MoS_2_-PEG nanoflakes after 6 cycles of laser irradiation does not show any appreciable change than that of the nanoflakes without laser irradiation, and the nanaoflakes also keep their colloidal stability after continuous 6 cycles of laser irradiation (insert of Fig. 3F). These combined results indicated that the synthesized MoS_2_-PEG nanoflakes possessed desirable photothermal stability under laser irradiation, which is thus beneficial for cancer PTT.

### *In vitro* cytotoxicity of MoS_2_-PEG nanoflakes

**Figure 4:**
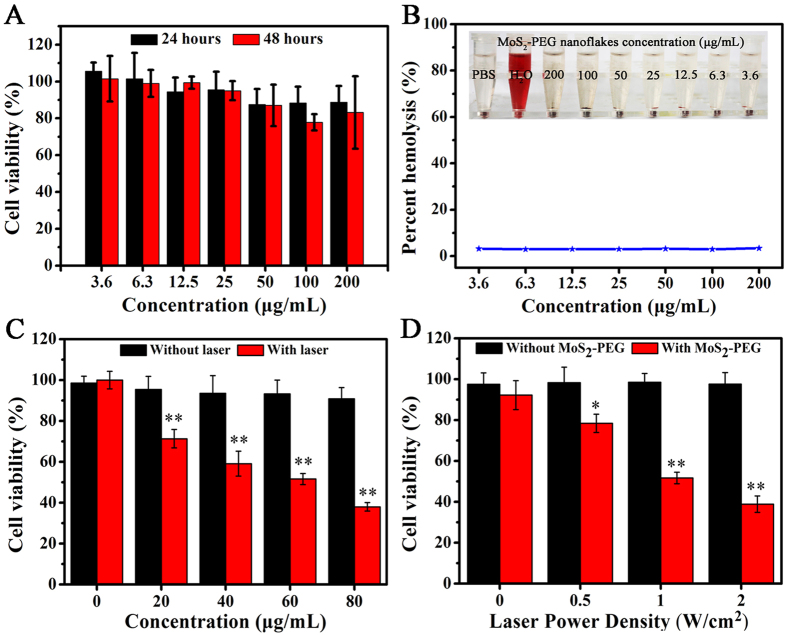
(**A**) Viabilities of 4T1 cells were estimated by the CCK-8 proliferation method versus incubation concentrations (3.6, 6.3, 12.5, 25, 50, 100 and 200 μg/mL) of the solutions of MoS_2_-PEG nanoflakes. Cells were incubated with the solution of MoS_2_-PEG nanoflakes at 37 °C for 24 and 48 h and treated with PBS were used as control. Data presented as mean ± standard deviation (n = 5). (**B**) Hemolytic percent of RBCs treated with MoS_2_-PEG nanoflakes with different concentrations, using deionized water (+) and PBS (−) as positive and negative controls, respectively. Inset: Photographs for direct observation of hemolysis. (**C**) The cell viability assay of 4T1 cells after exposed to different concentrations of MoS_2_-PEG nanoflakes with or without the irradiation of NIR 808-nm laser (1 W/cm^2^, 10 min). (**D**) Cell viability assay of 4T1 cells after treated with or without MoS_2_-PEG nanoflakes (60 μg/mL) under the irradiation of different NIR 808-nm laser power density. Data presented as mean ± standard deviation (n = 5).

The biocompatibility of the MoS_2_-PEG nanoflakes is the prerequisite for their practical biomedical applications. Cell Counting Kit-8 (CCK-8) assay was used to quantitatively determine the cytotoxicity of nanoflakes toward 4T1 cell. As presented in Fig. 4A, although cell viability was slightly reduced in a dose- and time-dependent manner, no significant cytotoxicity was observed after 24 and 48 h incubation of cells with MoS_2_-PEG nanoflakes at any concentration. Even after 48 h exposure to the highest concentration of nanoflakes (200 μg/mL), the viability of the cell population is more than 80%, indicating a very low cytotoxic effect of the MoS_2_-PEG nanoflakes.

It has been accepted that cell uptake of photothermal agents is desirable, because the intracellular photothermal agents can enhance the efficiency of photothermal cancer therapy^23^. Thus, to verify whether MoS_2_-PEG nanoflakes can be internalized by cancer cells, we quantitatively evaluated the cell uptake capability of MoS_2_-PEG nanoflakes on Murine breast cancer cells (4T1 cells) by using Inductively Coupled Plasma Atomic Emission Spectrometer (ICP-AES). The cellular-uptake study shows that MoS_2_-PEG nanoflakes facilitate the cell uptake and the cellular uptake is concentration-dependent (Supplementary Fig. S1). This is in good agreement with previous study that MoS_2_-PEG nanoflakes exhibited good dispersity and stability, which is beneficial to be taken up by cancer cells^23^. While maybe the particle size of nanoflakes is relatively large (in the range of 100–200 nm), which lead to the low cellular uptake amount of MoS_2_-PEG nanoflakes.

### Hemocompatibility of MoS_2_-PEG nanoflakes

Hemocompatibility of nanomaterials has been considered to be essential for their biomedical applications. Hemolysis assay, which establishes the interaction of nanomaterials with the integrity of Red blood cells (RBCs) membranes, yielded the main indicator of hemocompatibility. Therefore, we investigated the influence of MoS_2_-PEG nanoflakes on RBCs by using hemolysis assay. The hemolysis percentages results (Fig. 4B) showed that the hemolysis percentages of particles are all less than 4% even at the high concentration of 200 μg/mL. The results also can be detected from the optical image of blood samples where almost no hemolysis of RBCs was observed at the concentrations of MoS_2_-PEG nanoflakes ranging from 3.6 to 200 μg/mL, indicating that MoS_2_-PEG nanoflakes possess admirable hemocompatibility.

### Photothermal ablation of cancer cells *in vitro*

To verify the potential of MoS_2_-PEG nanoflakes as photothermal agents, the *in vitro* photothermal therapeutic efficiency against 4T1 cells was further investigated. Quantitative analysis of photothermal therapeutic efficiency was carried out after NIR 808-nm irradiation (1 W/cm^2^, 10 min) the 4T1 cells with different concentrations (0, 20, 40, 60 and 80 μg/mL) of MoS_2_-PEG nanoflakes for 10 min. As shown in Fig. 4C, 4T1 cells with MoS_2_-PEG nanoflakes showed significantly lower cell viability under 808-nm NIR laser irradiation compared with corresponding control experimental samples without NIR laser irradiation. Moreover, when simultaneously treatment with 80 μg/mL of MoS_2_-PEG nanoflakes and NIR laser irradiation (808 nm, 1 W/cm^2^, 10 min), the cell viability was significantly decreased to 38% after 24 h incubation, while more over 90% of cells viability remained alive without irradiation. In addition, the photothermal therapeutic efficiency was examined after treating the 4T1 cells with same concentration of MoS_2_-PEG nanoflakes under different laser power density (0, 0.5, 1 and 2 W/cm^2^). Thereafter, the 4T1 cells were incubated with 60 μg/mL of the MoS_2_-PEG nanoflakes and then exposed to laser radiation at different output power densities for 10 mins. As shown in Fig. 4D, 4T1 cells under NIR laser irradiation showed obviously lower cell viability treatment with MoS_2_-PEG nanoflakes than the corresponding control experimental samples without MoS_2_-PEG nanoflakes. When simultaneously treatment with MoS_2_-PEG nanoflakes (60 μg/mL) and NIR irradiation (808 nm, 2 W/cm^2^, 10 min), the cell viability was significantly decreased to 38.9% after 24 h incubation. While cells irradiated with 808 nm NIR laser (2 W/cm^2^, 10 min) without MoS_2_-PEG nanoflakes incubation had a cell viability of 97%. These results indicated that the combination of MoS_2_-PEG nanoflakes and NIR lase irradiation could induce localized hyperthermia to ablate the cancer cells *in vitro*.

**Figure 5:**
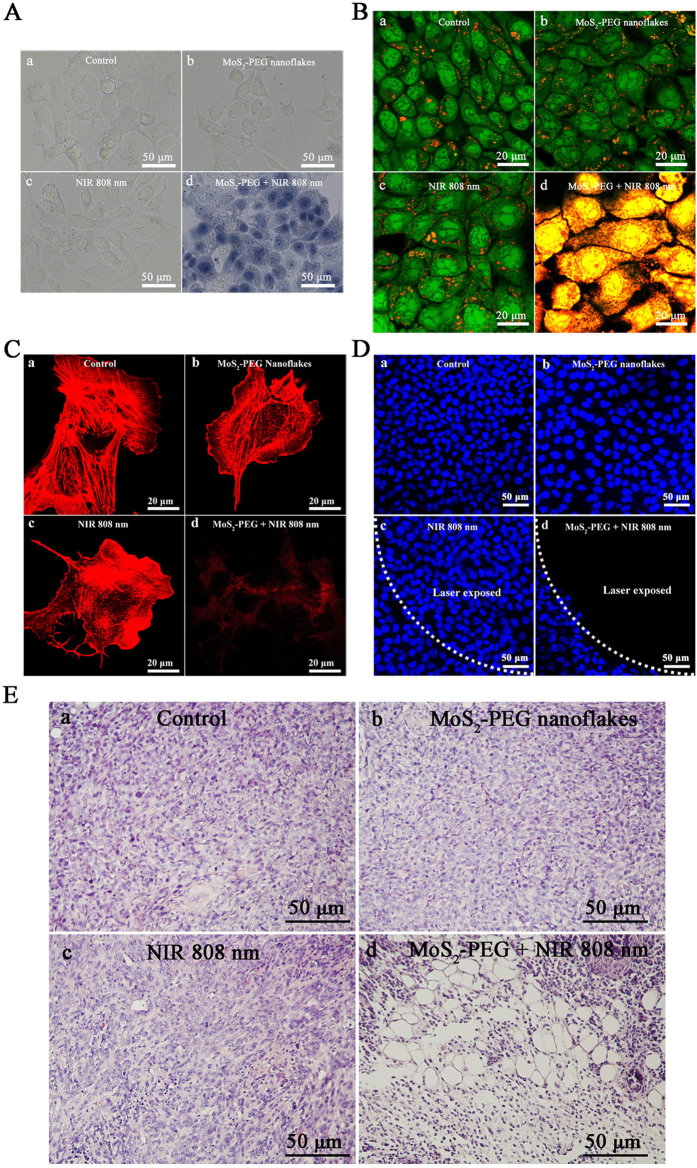
(**A**) Optical microscopy images of trypan blue stained 4T1 cells after incubation with different conditions as denoted in each individual image. Only destruction or dead cells can be stained to be blue. (**B**) Confocal laser scanning microscopy (CLSM) images of acridine orange (AO) stained 4T1 cells after incubation with different conditions as denoted in each individual picture. (**C**) CLSM images of Alexa Fluor®568 conjugated phalloidin (red) stained cells cytoskeleton after incubation with different conditions as denoted in each individual picture. (**D**) CLSM images of DAPI (blue) stained cell nuclei for clear observation after cells incubation with different conditions as denoted in each individual picture. (**E**) Representative H&E stained histological images of corresponding tumor sections from the tumor with different treatments. (a) Blank control (neither MoS_2_-PEG nanoflakes nor the NIR-laser treatment). (b) Only MoS_2_-PEG nanoflakes without NIR 808-nm laser was used treatment with 4T1 cells. (c) Only NIR 808-nm laser without MoS_2_-PEG nanoflakes was used treatment with 4T1 cells. (d) MoS_2_-PEG nanoflakes treated cells are irradiated by NIR 808-nm laser.

In PTT, NIR laser is used to excite the nanomaterials and create local temperature increase to destroy cancer cells. A living cell is a very complicated architecture consisting of diverse biomacromolecules, including proteins, lipids and gene, and thermally induced protein denaturation is considered the main factor causing cell/tissue death or injury in PTT^25^,^50^. We thus first evaluated the photothermal cytotoxicity of MoS_2_-PEG nanoflakes with and without laser irradiation against 4T1 cells using trypan blue assay. As seen from the optical images in Fig. 5A, all the cells remain alive without trypan blue staining upon treatment with MoS_2_-PEG nanoflakes or laser irradiation alone (Fig. 5Ab,c), whereas most 4T1 cells are destroyed as evident by the increased trypan blue staining when treated with MoS_2_-PEG nanoflakes (60 μg/mL) under laser irradiation at 2 W/cm^2^ for 5 min (Fig. 5Ad), thus creating a significant photothermal therapeutic effect for cancer cells.

To further evaluate *in vitro* therapeutic efficacy of the MoS_2_-PEG nanoflakes, the relative cell viabilities of HeLa cells upon different treatments were performed by CCK-8 assay (Supplementary Fig. S2A). When compared with control cells, the cell viability was not decreased significantly after treatment with nanoflakes or laser irradiation alone, which was also consistent with the cytotoxicity results showing MoS_2_-PEG nanoflakes to be very low cytotoxic to cells. By contrast, when simultaneously treatment with MoS_2_-PEG nanoflakes (60 μg/mL) plus laser irradiation (808 nm, 2 W/cm^2^, 10 min), the viability of HeLa cells was remarkably decreased to about 50% after 24 h incubation, suggesting a significant photohyperthermia effect for HeLa cells *in vitro*.

Subsequently, cells survival was then detected by staining with both acridine orange (AO) and propidium iodide (PI). Fluorescence microscopy images showed that HeLa cells treated with MoS_2_-PEG nanoflakes or lasers irradiation alone exhibited bright green colour without red fluorescence were observed, indicating the exposure of HeLa cell to either MoS_2_-PEG nanoflakes or NIR laser alone did not compromise cell viability. It was clearly seen that there was no apparent change in cell viability and density was observed when cells were treated with laser or MoS_2_-PEG nanoflakes alone (Supplementary Fig. S2Bb,c), well consistent with the CCK-8 assay results. In comparison, HeLa cells treated with MoS_2_-PEG nanoflakes plus a NIR 808 nm laser experienced substantial cellular death, as indicated by intense homogeneous red fluorescent (Supplementary Fig. S2Bd), suggesting that MoS_2_-PEG nanoflakes could mediate the photothermal destruction of HeLa cells.

### Effect of photothermal treatment on the lysosomal membrane integrity

Photothermal damage on cancer cells has been associated with the disruption of subcellular organelles such as lysosomes. Furthermore, for the sake of demonstrating the disruption, we further utilized AO as an intracellular marker, which can trace the integrity of lysosomal membrane. AO can emit red fluorescence in the intact acidic lysosomes and display green fluorescence in neutralized cytosol and nuclei. As the confocal lasers scanning microscopy (CLSM) images in Fig. 5B show, there was no detectable destabilization of lysosomal membranes in the 4T1 cells when treated with combined MoS_2_-PEG nanoflakes or laser irradiation alone, which was similar to the control group where some red fluorescence signal was observed. It indicated that the lysosomal compartments are still stable. However, after cells were treated with combined MoS_2_-PEG nanoflakes and laser irradiation, the CLSM image in Fig. 5Bd demonstrates that the red fluorescence was colocalized with green fluorescence of Lysotracker to yield orange-red staining throughout the entire cell. It indicates that the MoS_2_-PEG nanoflakes combined with NIR laser irradiation can effectively destroy the lysosomal membranes and cause a rapid leakage of AO staining of acidic organelles, which will lead to acute cell death. The disruption of lysosomal membranes is possibly attributed to the hyperthermia triggered by photothermal effect^51^. This finding establishes a clear link between the disruption of lysosomal integrity and the induction of cell apoptosis by photothermal effect.

### Effect of photothermal treatment on the cell cytoskeleton and cell adhesion

The filamentous actin (F-actin) cytoskeleton is a dynamic structure necessary for regulating cell functions such as cell apoptosis and internal architecture^52^,^53^. The photothermal ablation of cells may result in cytoskeleton disruption and cellular membrane dysfunction due to intracellular hyperthermia. The change in cytoskeleton organization of 4T1 cells treated with MoS_2_-PEG nanoflakes with and without laser irradiation was further investigated using fluorescent staining. Figure 5C showed the representative fluorescent images of F-actin stained with Alexa Fluor® 568 conjugated phalloidin after cells were subjected to various treatments. After cells were treated with MoS_2_-PEG nanoflakes or laser irradiation alone, the actin filaments were well-organized in thick bundles forming stress fibers. These fibers were stretched between cell upper surface and cytoplasm, indicative of cortical fibers. There was no significant difference between control cells and cells with above treatments. However, in the case of cells treated with MoS_2_-PEG nanoflakes under laser irradiation, the integrity of the cytoskeleton was disturbed as indicated by disruption of actin stress fibers, compared to control cells where the actin stress fibers were aligned in parallel bundles.

To examine how cell adhesion was affected by laser-induced photothermal effect and delineate the *in vitro* localized photothermal destruction of cancer cells, 4T1 cells were treated with different conditions and then incubated for 24 h. DAPI was used to stain cell nuclei for clear observation by confocal microscopy. As shown in Fig. 5D, 4T1 cells treated with either MoS_2_-PEG nanoflakes or laser irradiation alone showed blue fluorescence in the entire well (Fig. 5Da–c), suggesting the survival of 4T1 cells before DAPI staining. The white line shows the approximate location of the laser during exposure. Significantly, a big dark region that indicated the exfoliated area of death cell was observed only in the presence of both MoS_2_-PEG nanoflakes and laser irradiation, thus further demonstrating the photothermal ablation efficacy of MoS_2_-PEG nanoflakes under NIR laser irradiation (Fig. 5Dd). The dark region matched well with the location of laser irradiation on the well (the laser beam spot was 25 mm^2^). These results clearly demonstrated the feasibility of MoS_2_-PEG nanoflakes as PTT agents.

### Photothermal ablation of cancer cells *in vivo*

To shed more light on the photothermal effect of the MoS_2_-PEG nanoflakes, we further performed photothermal effect *in vivo*. The histological examination of tumors was performed by means of microscopic imaging (Fig. 5E). In the case of mice treatment with MoS_2_-PEG nanoflakes alone or NIR laser irradiation alone, there are no obvious differences regarding the cellular size and shape, nuclear modifications, or necrosis (Fig. 5Eb,c). Inspiringly, relative to an blank control with only saline injection (Fig. 5Ea), in the case of MoS_2_-PEG nanoflakes-injected mice, histological examination of the tumors treated with showed common signs of thermal cell damage under 1 W/cm^2^ NIR 808 nm laser irradiation, such as loss of contact, shrinkage of the cells, pyknotic and fragmentized nuclei (Fig. 5Ed). Taken together, these results unambiguously proof photothermal effects of MoS_2_-PEG nanoflakes.

In summary, we have synthesized the flower-like PEGylated MoS_2_ nanoflakes by a simple method and demonstrated its effectiveness as photothermal agent for cancer cell ablation. The synthesized MoS_2_-PEG nanoflakes exhibited good dispersion stability under different conditions, and achieved a very high photothermal conversion efficiency of 27.6% as well as desirable photothermal stability upon NIR laser irradiation. In addition, the MoS_2_-PEG nanoflakes displayed a very low cytotoxicity on cells even at the highest tested concentration of 200 μg/mL. Furthermore, *in vitro* photothermal anticancer activity results demonstrated that an effective photothermal killing of cancer cells could be realized by a low concentration of nanoflakes under a low power 808 nm laser irradiation. Our results also demonstrated that the photothermal treatment of cancer cells could damage the integrity of lysosomal membrane and cell skeleton and therefore efficiently kill cancer cells. Moreover, cancer cells *in vivo* could be efficiently destroyed by the photothermal effects of MoS_2_-EPG nanoflakes under the irradiation of NIR 808 nm laser. Although further cellular and *in vivo* studies are actually required, the synthesized MoS_2_-PEG nanoflakes clearly showed good colloidal and photothermal stability, very low cytotoxicity and effective photothermal ablation of cancer cells, which would be promising materials for future PTT applications.

## Methods

### Materials

Ammonium tetrathiomolybdate ((NH_4_)_2_MoS_4_), hydrazine hydrate (N_2_H_4_·H_2_O, 98%) and alpha-lipoic acid (LA) were purchased from J&K Scientific Ltd (Beijing, China). Methoxy-poly(ethylene glycol)-amine (mPEG-NH_2_) was purchased from Shanghai Yarebio Co., Ltd. (Shanghai, China). N,N’-dicyclohexylcarbodiimide (DCC) and trimethylamine (TEA) were obtained from Sigma-Aldrich (Shanghai) Trading Co., Ltd (Shanghai, China). Bovine serum albumin (BSA), hematoxylin and eosin (H&E) and Triton® X-100 were obtained from Sigma-Aldrich (Shanghai) Trading Co., Ltd (Shanghai, China). All cell-culture related reagents such as RPMI 1640 medium, FBS, trypsin and penicillin-streptomycin were purchased from Thermo Scientific HyClone (Beijing, China). CCK-8 and trypan blue were purchased from Beyotime Institute of Biotechnology (China). (4′,6-diamidino-2-phenylindole) (DAPI), AO and PI were purchased from BestBio Biotechnology Co., Ltd (Shanghai, China). Paraformaldehyde was obtained from Shanghai Suolaibao Bio-Technology Co., Ltd. (Shanghai, China). Alexa Fluor® 568 conjugated phalloidin was obtained from Molecular Probes (Invitrogen, USA). DI water (18.2 mΩ cm resistivity) was used for sample washing and solution preparation throughout the experiments. All other chemical reagents were of analytical grade and used as received.

### Preparation of MoS_2_ nanoflakes

MoS_2_ nanoflakes were synthesized as previously described method with some modification^54^. In a typical procedure, 27.5 mg of (NH_4_)_2_MoS_4_ powder was dispersed in 12.5 mL of H_2_O. After stirring 20 min and bath sonication for 10 min until homogeneous, 0.125 mL of N_2_H_4_·H_2_O was added into the aforementioned solution and was further bath sonicated for 30 min. The mixture was then transferred into a 50 mL Teflon-lined stainless steel autoclave, sealed tightly and heated in an oven at 200 °C for 10 h. After cooling down naturally to room temperature, the black product was collected by centrifugation at 10^4^ rpm for 5 min, washed thoroughly with DI water to remove unreacted reagents and N_2_H_4_·H_2_O residuals, and recollected by centrifugation. The washing step was repeated for at least 10 times to ensure that the unreacted reagents were removed. The product was redispersed and dialyzed against DI water to completely remove residue ions. Finally, the purified product was dispersed in 5 mL DI water for future use.

### Synthesis of the LA-PEG precursors

LA-PEG polymer was synthesized according to a reported approach with slight modification^55^. Briefly, 54 mg of lipoic acid was dissolved with stirring in 2.4 mL of dichloromethane (DCM), and then 600 mg of mPEG-NH_2_ (Mw = 5000) was added with stirring until the solution was clear. After stirring 24 h, 12 mg of DCC and 7.2 μL of TEA were added and stirred for 2 h. The solvent was removed by rotary evaporation and the obtained crude product was added to 10 mL of DI water. After the insoluble by-product was removed by filtration, the filtrate was adjusted to pH 8.0 with 0.1 M sodium bicarbonate. The product of LA-PEG was collected by extraction with DCM for 3 times, and dried for further use.

### Synthesis of the MoS_2_-PEG nanoflakes

For the PEG functionalization, 15 mg of LA-PEG was dispersed into the prepared 3 mL of MoS_2_ nanoflakes aqueous solution (0.5 mg/mL). The obtained suspension was then treated with bath sonication for 30 min. After stirring another 12 h, MoS_2_-PEG nanoflakes were collected by centrifugation and washed thoroughly several time with DI water to remove excess LA-PEG polymer. The MoS_2_-PEG nanoflakes were resuspended in DI water and stored at 4 °C before future use.

### Photothermal performance measurement of MoS_2_-PEG nanoflakes

The photothermal heating performance of MoS_2_-PEG nanoflakes was carried out by a laser light source equipped with an external adjustable power (0–5 W/cm^2^) 808-nm continuous-wave NIR laser device (Tours Radium Hirsh Laser Technology Co., Ltd., Xi’an, China). The output power was independently calibrated using a handheld model 1918-C optical power meter (Newport Corp. CA, USA). The individual of MoS_2_-PEG nanoflakes stock solution at 200 μg/mL was diluted to different concentrations (5, 20, 40, 60 and 80 μg/mL) and 0.4 mL of aqueous dispersion was introduced in a quartz cuvette. The 808 nm NIR laser was employed to deliver perpendicular through the aforementioned quartz cuvette. The temperature of the solutions was monitored every 20 seconds by using a thermocouple thermometer (DT-8891E, Shenzhen Everbest Machinery Industry Co., Ltd, China) with a thermocouple probe (accuracy: ±1 °C).

The photothermal conversion efficiency (*η*) of MoS_2_-PEG nanoflakes was accurately measured. The sample dispersions were first irradiated with 808-nm NIR laser at a power of 2 W/cm^2^, followed by naturally cooling down to the room temperature without laser irradiation. The 808-nm NIR laser induced temperature change of the MoS_2_-PEG nanoflakes dispersion was monitored as a function of time under continuous irradiation until a steady-state temperature was reached. The *η* value could be calculated by the following equation^56^.


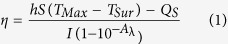


Where the following parameters are defined: the photothermal conversion efficiency *η*, the heat transfer coefficient *h*, the surface area of the sample cuvette *S*, the steady-state temperature *T*_*max*_, the temperature of the surroundings *T*_*Sur*_, the heat associated with the light absorbance of the solution *Q*_*S*_, the incident laser power *I*, and the absorbance at a wavelength of 808 nm of the MoS_2_-PEG nanoflakes *A*_*λ*_.

### Cell line and cell culture condition

4T1 cells and HeLa cells were obtained from Chinese Academy of Sciences Cell Bank for Type Culture Collection (Shanghai, China). 4T1 cells were routinely cultured in complete RPMI 1640 medium containing 10% (v/v) FBS, 1% (v/v) 100 U/mL penicillin and 100 μg/mL streptomycin. HeLa cells were cultured in DMEM with 10% FBS, 100 U/mL penicillin and 100 μg/mL streptomycin. All the cells were grown in a humidified incubator at 37 °C under atmosphere supplemented with 95% air and 5% CO_2_. The entire medium was changed every day, and cells were always trypsinized and harvested before reaching confluence so that they were never subject to crowded conditions.

### *In vitro* photothermal ablation of MoS_2_-PEG nanoflakes for cancer cells

For quantitatively analyze the *in vitro* PTT anticancer effects of MoS_2_-PEG nanoflakes, 4T1 cells with a density of 10^4^ cells per well were seeded into a 96-well plate for 24 h to allow cell attachment. Thereafter, the culture medium was removed and the cells were treated with MoS_2_-PEG nanoflakes aqueous dispersions at various desired concentrations (0, 40, 40, 60 and 80 μg/mL) at 37 °C for 2 h. The cells were then treated with or without irradiation of NIR 808-nm laser at different out power densities for 10 min. After irradiation treatment, the cells were then incubated at 37 °C for another 24 h. CCK-8 assay was performed to evaluate the cell viabilities.

Trypan blue staining was used to evaluate the viability of 4T1 cells. 4T1 cells were first seeded into 20-mm glass bottom culture dishes at a density of 2 × 10^4^ cells per well and incubated for 24 h at 37 °C. Thereafter, the culture medium was removed, and cells were randomly divided into four groups: group I, blank control cells; group II, MoS_2_-PEG nanoflakes (60 μg/mL) alone; group III, NIR 808-nm treatment only; and group IV, MoS_2_-PEG nanoflakes (60 μg/mL) + NIR 808-nm. At the end of incubation for 2 h, the cells of group III and IV were exposed to an 808-nm laser at a power density of 2.0 W/cm^2^ for 10 min. The culture medium was discarded and 4T1 cells were then rinsed twice with PBS. All the cells were incubated with a dilution of the 0.4% (w/v) trypan blue solution in PBS for 5 min to test cell viability. After washed more than three times by PBS, cells were observed on a light microscope (Olympus, BH-2) in bright field. Each experiment was repeated three times and representative results are shown. Injured or dead cells accumulated the dye and were stained blue, while live cells could pump it out and remain clear.

### Effect of photothermal treatment on the lysosomal membrane integrity

The AO staining method was used to investigate the integrity of lysosomal membrane. 4T1 cells were randomly divided into four groups: group I, blank control cells; group II, MoS_2_-PEG nanoflakes (60 μg/mL) alone; group III, NIR 808-nm treatment (2.0 W/cm^2^, 10 min) only; and group IV, MoS_2_-PEG nanoflakes (60 μg/mL) + NIR 808-nm (2.0 W/cm^2^, 10 min). For AO staining, culture medium was discarded and 4T1 cells were rinsed twice with PBS. Then 5 μg/mL of AO in complete medium was added to each dish at 37 °C for further incubation 15 min. Before cells were observed by a Carl Zeiss LSM 700 CLSM (He-Ne and Ar lasers), the cells were washed with PBS again and serum-free medium was added into the wells. AO was excited at 488 nm, and emission signals were detected at 530 nm (green color) and 590 nm (red color).

### Effect of photothermal treatment on the cell cytoskeleton

To assess the effect of MoS_2_-PEG nanoflakes on cell cytoskeleton, 4T1 cells were seeded and divided into four groups as described above. After different treatment, all the cells were further incubated at 37 °C for 12 h. The culture medium was replaced by preheated PBS buffer and fixed with fresh 4.0% paraformaldehyde at room temperature for 20 min. After rinsing with PBS, the cell samples were permeabilized with 0.1% Triton® X-100 in PBS for 5 min and blocked with 1% BSA in PBS for 30 min. The cells were further washed with PBS, and were subjected to F-actin staining with Alexa Fluor® 568 conjugated phalloidin for 30 min before confocal imaging.

### Photothermal ablation of cancer cells *in vivo*

Female Balb/c mice were purchased from Shanghai Slac Laboratory Animal Co., Ltd. (Shanghai, China). All animal experiments were performed in compliance with the Institutional Animal Care and Use Committees (IACUC) guidelines. The tumors were generated by subcutaneous injection of 2 × 10^6^ 4T1 cells on the back of each Balb/c mice. When tumors grew to 3–6 mm in diameter, the Balb/c mice were randomly assigned into four groups, which were labeled as control and treatment groups (n = 3 per group): group I, blank control mice; group II, MoS_2_-PEG nanoflakes treatment alone; group III, NIR 808-nm treatment only; and group IV, MoS_2_-PEG nanoflakes + NIR 808-nm. The Balb/c mice were first anaesthetized by pentobarbital sodium at a dosage of 50 mg/kg body weight, and then the treatment and control samples were injected with 100 μL of PBS solution containing the MoS_2_-PEG nanoflakes (80 μg/mL) and saline, respectively, at the central region of the tumors with a depth of ~3 mm. After 1 h, the mice with tumors of the group III and IV were irradiated with 808 nm laser (an output power density of 1 W/cm^2^) for 15 min. The Balb/c mice were scarified and collected the tumors. After rinsed three times with physiological saline, each tumor was soon immersed in 10% formalin for pathology analysis. After immersed for 48 h, the tissues were embedded in paraffin, sectioned into 4 μm thick sections, stained with H&E and examined by light microscope.

### Statistical analysis

All quantitative data were expressed as the mean ± standard deviation (S.D). One-way analysis of variance (one-way ANOVA) and Scheffe’s post hoc test were used for statistical analyses. The statistical significance for all tests were set at *P < 0.05 and **P < 0.01.

## Additional Information

**How to cite this article**: Feng, W. *et al.* Flower-like PEGylated MoS_2_ nanoflakes for near-infrared photothermal cancer therapy. *Sci. Rep.* **5**, 17422; doi: 10.1038/srep17422 (2015).

## Supplementary Material

Supplementary Information
