# Supplementary material for: Flower-like PEGylated MoS2 nanoflakes for near-infrared photothermal cancer therapy
Source: Sci Rep. 2015 Dec 3;5:17422. doi: 10.1038/srep17422 (PMC4668368; doi:10.1038/srep17422)
Supplement: Supplementary Information [file srep17422-s1.pdf]

Supplementary Information:

Flower-like PEGylated MoS_2 nanoflakes for near-infrared photothermal cancer therapy

Wei Feng,[1] *Liang Chen,*[1] *Ming Qin,*[1] *Xiaojun Zhou,*[2] *Qianqian Zhang,*[1] *Yingke Miao,*[1] *Kexin Qiu,*[2] *Yanzhong Zhang*[1] *and Chuanglong He*[*, 1]

[1] College of Chemistry, Chemical Engineering and Biotechnology, Donghua University, Shanghai 201620, China

[2] College of Materials Science and Engineering, Donghua University, Shanghai 201620, China

*Corresponding author.

Professor Chuanglong He

College of Chemistry, Chemical Engineering and Biotechnology, Donghua University, 2999 North Renmin Road, Shanghai 201620, China.

Tel. /fax: +86 21 6779 2742

Email address: hcl@dhu.edu.cn (C.L He)

Characterization. The morphologies and microstructures of the synthesized samples were examined by a Hitachi S-4800 field emission scanning electron microscope (FESEM, Hitachi Ltd., Japan) and a JEM-2100 transmission electron microscope (TEM, JEOL Ltd., Japan) with acceleration voltage of 200 kV. The size distribution of nanoflakes was carried out by dynamic light scattering (DLS) method using a BI-200SM multi-angle dynamic/static laser scattering instrument (Brookhaven, USA). Zeta-potential analyses were measured using a Zetasizer Nano ZS apparatus (Malvern Instruments, UK). The Fourier transform infrared (FTIR) spectrum was recorded by KBr pellet technique using a Nexus 670 spectrometer (Thermo Nicolet, USA). Ultraviolet-visible (UV-vis) absorption spectra were measured at room temperature on a Lambda 35 UV-vis spectrophotometer (PerkinElmer, USA). Thermogravimetric (TG) curves were recorded on a TG 209 F1 analyzer (Netzsch, Germany) under air flow (50 mL/min) from the room temperature to 900 °C with a ramp of 10 °C/min. The X-ray photoelectron spectroscopy (XPS) was recorded by using a PHI 5300 ESCA XPS (Philadelphia, USA) spectrometer with monochromatic Mg-K_α radiation as the excitation source.

***In vitro* cytotoxicity assay and cellular uptake of MoS_2-PEG nanoflakes.** The MoS_2 has a strong absorption in the range of 400-600 nm, which is overlap with the absorption band of Cell Counting Kit-8 (CCK-8) solution. So the *in vitro* cytotoxicity of MoS_2-PEG nanoflakes against 4T1 cells was performed by the standard CCK-8 assay with slight modification[1,2]. Briefly, 4T1 cells were placed in 96-well plates at a density of 10^4 viable cells per well and cultured for 24 h to allow cell attachment.

Afterward, the cells were treated with MoS_2-PEG nanoflakes aqueous dispersions at various concentrations (0, 12.5, 25, 50, 100 and 200 μg/mL) at 37 °C for 24 and 48 h. A culture medium without nanoflakes was used as the blank control. After discarding the medium with or without MoS_2-PEG samples, the cells were washed twice with phosphate buffer saline (PBS) to remove the residual MoS_2-PEG, 90 μL fresh RPMI 1640 serum-free medium containing 10 μL CCK-8 solutions was added and the cells were incubated for another 2 h. The absorbance of each well was monitored at 450 nm on a microplate reader (MK3, Thermo, USA). The results of cytotoxicity were expressed as the percentage of cell viability. The relative cell viability was expressed as the Equation 2, and five parallel experiments were carried out for each treatment group.

$$Cell\ viability\ (\%) = \frac{A_{sample}}{A_{Control}} \times 100\% \qquad (1)$$

Where A_{Sample} and $A_{Control}$ are the absorbance of cell treated with and without MoS_2-PEG nanoflakes, respectively.

For cellular uptake, 4T1 cells were seeded in 6-well plates (3×10^5 cells per well) and incubated for 24 h. Then the old medium was removed and the 4T1 cells were cultured with fresh RPMI-1640 medium containing MoS2-PEG nanoflakes at the concentration of 40 and 80 μg/mL at 37 °C for 12 h. After discarding the cell culture medium, the cells was gently rinsed with prewarmed PBS, harvested by trypsinization, fixed with 2.5% glutaraldehyde and dehydrated in a graded series of ethanol (30, 50, 60, 70, 80, 90 and 100%) for 15 min each. The 4T1 cells were collected by

centrifugation, vacuum dried, weighted and treated with 1 mL aqua regia solution (HCl : HNO_3= 1 : 3, volume ratio) for 2 h. Finally, the cellular uptake of Mo content was determined by using an Inductively Coupled Plasma Atomic Emission Spectrometer (ICP-AES, Leeman, Prodigy).

***In vitro* hemolysis assay.** To investigate the *in vitro* hemocompatibility, 2 mL of fresh blood samples were obtained from mice and stabilized with heparin anticoagulants. Red blood cells (RBCs) were first separated from whole blood by centrifugation at 3000 rpm for 10 min at 4 °C to remove the plasma, buffy coat and the top layer of cells. The RBCs were further purified five times with PBS. The purified RBCs were then diluted to 10 mL PBS. Subsequently, 0.2 mL of diluted RBCs suspension was taken out to add to 1.2 mL of MoS_2-PEG nanoflake suspensions with different concentrations from 0.05 to 200 μg/mL in PBS. Meanwhile, 1.2 mL of PBS or DI water was used as negative or positive control, respectively. Then, all the mixtures were gently shaken and incubated at 37 °C for 3 h, followed by centrifugation at 8000 rpm for 3 min. The absorbance values of the supernatants were measured by using a UV-vis spectrophotometer at 541 nm. The equation for calculating hemolysis percent of RBCs are shown below and the average value was reported as the mean of five parallel samples.

$$Hemolysis\ \% = \frac{A_{Sample} - A_{Negative\ control}}{A_{Positive\ control} - A_{Negative\ control}} \times 100\% \qquad (2)$$

Where A_{Sample}, $A_{Negative\ control}$, and $A_{Positive\ control}$ are the absorbance of samples, negative control (PBS) and positive control (H_2O), respectively.

***In vitro* photothermal ablation of MoS_2-PEG nanoflakes against HeLa cells.** HeLa cells were first plated in a 96-well plate at a density of 10^4 cells per well for 24 h to allow cell attachment. Thereafter, the culture medium was removed, and cells were divided into four groups: group I, blank control cells; group II, MoS_2-PEG nanoflakes (60 μg/mL) alone; group III, NIR 808-nm treatment only; and group IV, MoS_2-PEG nanoflakes (60 μg/mL) + NIR 808-nm. At the end of incubation for 2 h, the cells of group III and IV were exposed to an 808-nm laser at a power density of 2.0 W/cm^2 for 10 min. At the end of incubation for 2 h, the cells of group III and IV were exposed to an 808-nm laser at a power density of 2.0 W/cm^2 for 10 min. After irradiation treatment, all the cells were then incubated at 37 °C for another 24 h. CCK-8 assay was performed to evaluate the cell viabilities.

For acridine orange (AO)/propidium iodide (PI) staining, 4T1 cells were seeded and divided into four groups as described above. After different treatment, culture medium was discarded and 4T1 cells were rinsed twice with PBS. 300 μL of dye mixture containing 50 nM AO and 300 nM PI were then added to the wells. After incubation for 10 min, the sample was washed by PBS solution and the images of the labeled cells were observed immediately by using a fluorescence microscope (Olympus IX71). Each experiment was performed three times.

Cellular uptake of MoS_2-PEG nanoflakes

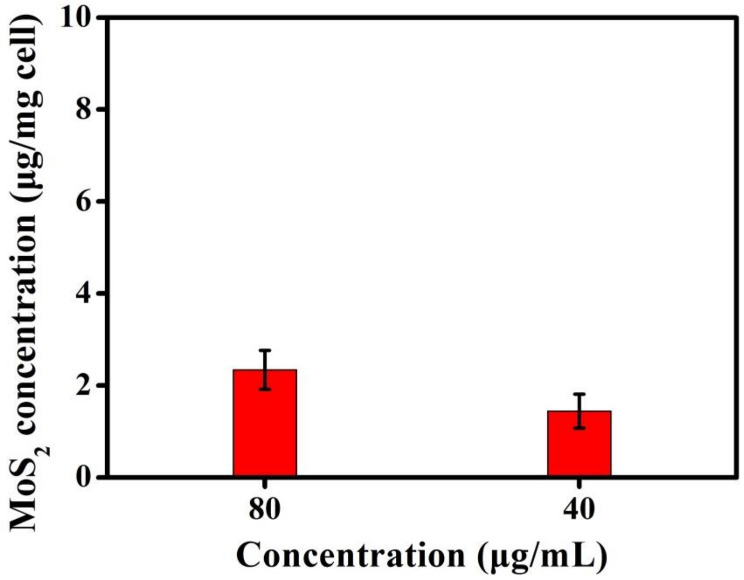

Fig. S1 Quantitative analysis of cellular uptake of MoS_2-PEG nanoflakes at 40 and 80 μg/mL for 12 h (mean ± SD, n=3).

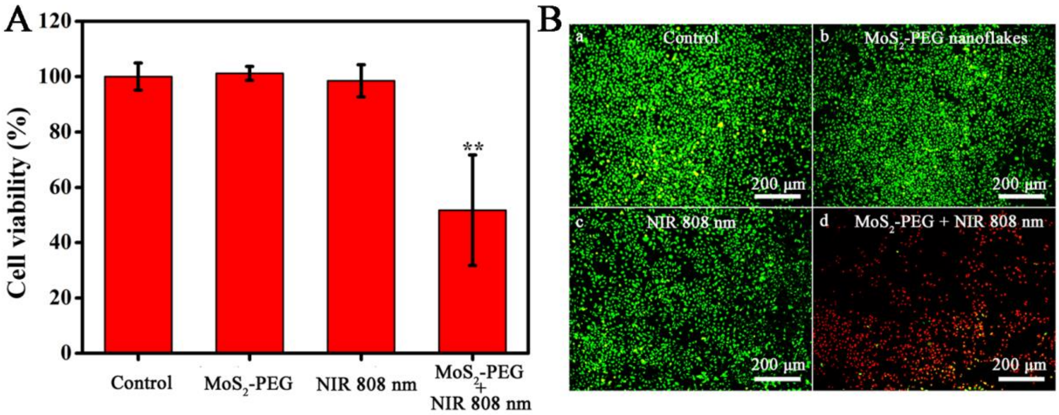

Fig. S2 (A) HeLa cell viability assay of MoS_2-PEG nanoflakes with different treatment. Data presented as mean ± standard deviation (n = 5) , ** $p<0.01$. (B) Fluorescence microscopy images of AO (green) and PI (red) co-staining of HeLa cells after incubation with different conditions as denoted in each individual picture. (a) Blank control (neither MoS_2-PEG nanoflakes nor the NIR-laser treatment). (b) Only MoS_2-PEG nanoflakes (60 μg/mL) without NIR 808-nm laser was used treatment with 4T1 cells. (c) Only NIR 808-nm laser (2 W/cm^2, 10 min) without MoS_2-PEG nanoflakes was used treatment with 4T1 cells. (d) MoS_2-PEG nanoflakes (60 μg/mL) treated cells are irradiated by NIR 808-nm laser with the power density of 2 W/cm^2 for 10 min.

Photothermal conversion efficiency calculation

The photothermal conversion efficiency (η) of MoS_2-PEG nanoflake was calculated according to the previously reported methods, detailed calculation as following:[3, 4]

During the photothermal heating process, the total energy balance for the system can be expressed as:

$$\sum_i m_i C_{P,i} \frac{d\Delta T}{dt} = Q_{NF} + Q_S - Q_{Loss} \quad (3)$$

Where m (g) represents the mass of the solution (m_s) and sample cuvette (m_q), C (J/(g·°C)) includes the constant-pressure heat capacity of solution (c_s) and sample cuvette (c_q), ΔT (°C) is the difference between the solution temperature T at time t and the starting solution temperature T_0, Q_{NF} (mW) is determined as the energy arising from the MoS_2-PEG nanoflakes, and Q_{Loss} (mW) is the thermal energy lost to the surrounding environment. In addition, Q_S (mW) is the energy input by the sample cuvette and the solvent (pure DI water), which is measured independently to be 9.0mW by using sample cuvette and pure water without MoS_2-PEG nanoflakes

For Q_{NF}, Equation (4) can be given as:

$$Q_{NF} = I(1 - 10^{-A_{808}})\eta \quad (4)$$

Where I = 2000 mW/cm^2 is the laser power which is incident on the system, A_{808} is defined as the absorbance of the MoS_2-PEG nanoflakes at the wavelength of 808 nm, and η is known as the photothermal conversion efficiency from the absorbed laser energy to thermal energy.

Furthermore, the energy dissipation mainly occurs through the heat conduction and thermal radiation. Q_{Loss} is linear with temperature for the outgoing thermal energy, then take the form as Equation (5):

$$Q_{Loss} = hS\Delta T = hS(T - T_{Sur}) \quad (5)$$

Where h (mW/(m^2·°C)) is heat transfer coefficient, S (m^2) is the surface area of the container, ΔT is the temperature change which is defined as T- T_{sur}, T (°C) is the water temperature and T_{Surr} (°C) is the solution temperature ambient temperature of surrounding environment.

When the temperature rises at a maximum steady-state temperature T_{Max} (°C), the system reaches the steady state. In this case, the heat input is equal to heat output, and the left side of Equation (3) becomes zero. So we then obtain

$$Q_{NF} + Q_S = Q_{Loss} = hS(T_{Max} - T_{Sur}) \quad (6)$$

Then η can be determined by combining Equation (3-6) and rearranging:

$$\eta = \frac{hS(T_{Max} - T_{Sur}) - Q_S}{I(1 - 10^{-A_{808}})} \quad (7)$$

Where Q_S is measured independently to be 9.0 mW, the (T_{Max}-T_{Sur}) is 46.7°C, I is 2000 mW/cm^2, A_{808} is 0.96. Thus, in the Equation (7), only the hS remains unknown parameter for calculating η.

In order to solve hS, the following notation θ is used herein, which is defined as the ratio of (T-T_{surr}) to (T_{Max}-T_{sur}):

$$\theta = \frac{T - T_{Sur}}{T_{Max} - T_{Sur}} \tag{8}$$

And a sample system time constant τ_s (s) is introduced:

$$\tau_s = \frac{\sum_i m_i C_{P,i}}{hS} \tag{9}$$

Substituting Equations (8) and (9) into Equation (3) and rearranging to obtain:

$$\frac{d\theta}{dt} = \frac{1}{\tau_s}\left[\frac{Q_{NF} + Q_S}{hS(T_{max} - T_{Sur})} - \theta\right] \tag{10}$$

When at the cooling stage of MoS_2-PEG nanoflakes aqueous dispersion, the laser source has been shut off, so the Q_{NF} + Q_S= 0. Under this condition, Equation (10) becomes:

$$dt = -\tau_s \frac{d\theta}{\theta} \tag{11}$$

Note that after integration Equation (11), the Equation expresses as:

$$t = -\tau_s \ln\theta \tag{12}$$

Therefore, time constant for heat transfer from the system is determined to be τ_s = 157s by applying the linear time data from the cooling period (after 300 s) *vs* –lnθ (Fig. 3D). In addition, the *m* is 0.4 g and the *C* is 4.2 J/g°C. Thus, according to Equation (9), the *hS* is calculated to be 10.7 mW/°C. Substituting *hS* = 10.7 mW/°C into Equation (7), the result photothermal conversion efficiency (η) of MoS_2-PEG nanoflakes can be calculated to be 27.6 %.

References:

1 Feng, W. *et al.* Polyelectrolyte multilayer functionalized mesoporous silica nanoparticles for pH-responsive drug delivery: layer thickness-dependent release profiles and biocompatibility. *J. Mat. Chem. B* **1**, 5886-5898, (2013).

2 Feng, W. *et al.* Effect of pH-Responsive alginate/chitosan multilayers coating on delivery efficiency, cellular uptake and biodistribution of mesoporous silica nanoparticles based nanocarriers. *ACS Appl. Mater. Interfaces* **6**, 8447-8460, (2014).

3 Roper, D. K., Ahn, W. & Hoepfner, M. Microscale heat transfer transduced by surface plasmon resonant gold nanoparticles. *J. Phys. Chem. C* **111**, 3636-3641, (2007).

4 Feng, W. *et al.* Au/Polypyrrole@Fe_3O_4 nanocomposites for MR/CT dual-modal imaging guided-photothermal therapy: An *in vitro* study. *ACS Appl. Mater. Interfaces* **7**, 4354-4367, (2015).
